# *In vivo* imaging of therapy response to a novel Pan-HER antibody mixture using FDG and FLT positron emission tomography

**DOI:** 10.18632/oncotarget.6060

**Published:** 2015-10-09

**Authors:** Carsten H. Nielsen, Mette M. Jensen, Lotte K. Kristensen, Anna Dahlman, Camilla Fröhlich, Helle J. Jacobsen, Thomas T. Poulsen, Johan Lantto, Ivan D. Horak, Michael Kragh, Andreas Kjaer

**Affiliations:** ^1^ Minerva Imaging ApS, Copenhagen, Denmark; ^2^ Department of Clinical Physiology, Nuclear Medicine and PET and Cluster for Molecular Imaging, Rigshospitalet and University of Copenhagen, Copenhagen, Denmark; ^3^ Symphogen A/S, Ballerup, Denmark

**Keywords:** PET/CT, FDG and FLT, HER family, antibody therapy, pancreatic cancer

## Abstract

**Purpose:**

Overexpression of the human epidermal growth factor receptor (HER) family and their ligands plays an important role in many cancers. Targeting multiple members of the HER family simultaneously may increase the therapeutic efficacy. Here, we report the ability to image the therapeutic response obtained by targeting HER family members individually or simultaneously using the novel monoclonal antibody (mAb) mixture Pan-HER.

**Experimental design and results:**

Mice with subcutaneous BxPC-3 pancreatic adenocarcinomas were divided into five groups receiving vehicle or mAb mixtures directed against either EGFR (HER1), HER2, HER3 or all three receptors combined by Pan-HER. Small animal positron emission tomography/computed tomography (PET/CT) with 2′-deoxy-2′-[^18^F]fluoro-D-glucose (FDG) and 3′-deoxy-3′-[^18^F]fluorothymidine (FLT) was performed at baseline and at day 1 or 2 after initiation of therapy. Changes in tumor uptake of tracers were quantified and compared to reduction in tumor size. Imaging results were further validated by immunohistochemistry and qPCR. Mean FDG and FLT uptake in the Pan-HER treated group decreased by 19±4.3% and 24±3.1%, respectively. The early change in FDG and FLT uptake correlated with tumor growth at day 23 relative to day 0. *Ex vivo* molecular analyses of markers associated with the mechanisms of FDG and FLT uptake confirmed the *in vivo* imaging results.

**Conclusions:**

Taken together, the study supports the use of FDG and FLT as imaging biomarkers of early response to Pan-HER therapy. FDG and FLT PET/CT imaging should be considered as imaging biomarkers in clinical evaluation of the Pan-HER mAb mixture.

## INTRODUCTION

Dysregulation and overexpression of the human epidermal growth factor receptor (HER) family and their ligands play an important role in many cancers. The HER family consists of four members: EGFR/HER1, ErbB-2/HER2, ErbB-3/HER3, and ErbB-4/HER4. Approved cancer treatments based on monoclonal antibodies (mAb) target receptors individually. However, it has recently been shown that simultaneously targeting EGFR, HER2, and HER3 with a Pan-HER (mAb) mixture consisting of two EGFR-, two HER2- and two HER3-targeting mAbs with non-overlapping epitopes in a single drug compound increases the therapeutic efficacy compared with targeting the receptors individually [1]. Previous studies have additionally shown that the human pancreatic cancer cell line BxPC-3 is dependent on more than one of the HER family members [2, 3]. Pan-HER is highly efficacious against this cancer cell line, and all three target specificities have been shown to contribute to the anti-proliferative activity *in vivo* [1].

Response Evaluation Criteria in Solid Tumors (RECIST) is frequently used for evaluation of therapeutic response [4]. In the RECIST guidelines, evaluation of treatment response is based on anatomical imaging with computed tomography (CT) or magnetic resonance imaging (MRI), which does not give information on the biological processes induced by the therapy. Moreover, morphological response is a late-occurring event. Development of predictive biomarkers of early response to therapy has gained much interest due to both their potential to accelerate the drug development process and their potential to differentiate responding from non-responding patients early after initiation of therapy. Positron emission tomography (PET) is an imaging technique that allows for non-invasive and longitudinal studies of biological function in intact living organisms. The PET tracers 2′-deoxy-2′-[^18^F]fluoro-D-glucose (FDG) and 3′-deoxy-3′-[^18^F]fluorothymidine (FLT) are used to measure tumor glucose uptake and tumor cell proliferation, respectively. The glucose analogue FDG is a widely used PET tracer for diagnosis and staging of cancer [5]. FDG enters the cell via the same mechanism as glucose, but once phosphorylated FDG accumulates due to no further metabolism. The thymidine analogue FLT enters the cells by the pyrimidine salvage pathway and phosphorylation of FLT by thymidine kinase 1 (TK1) results in intracellular trapping of FLT [6, 7]. Several studies have shown a positive correlation between FLT uptake and tumor cell proliferation [8-11]. PET imaging with FDG and FLT has previously shown promise in preclinical studies to monitor treatment response to therapies targeting different members of the HER family. Treatment of mouse models of human cancer with the EGFR targeting mAb cetuximab induced decreases in FLT uptake [12, 13]. Likewise, inhibition of EGFR with the small molecule inhibitor erlotinib reduced uptake of FLT [12, 14, 15]. Results from preclinical studies analyzing FDG uptake after EGFR inhibition are more variable. Following treatment initiation with erlotinib one study observed decreases in FDG uptake [16], whereas another study observed unchanged FDG uptake [14]. Inhibition of several members of the HER family simultaneously with the small molecules CI-1033 and PKI-166 induced decreases in FDG and FLT uptake [17, 18]. In contrast, treatment with afatinib, an inhibitor of HER1, HER2 and HER4, did not change FDG uptake [19]. In clinical studies, early FDG and FLT PET scans have been shown to predict progression-free survival after treatment with erlotinib [20, 21]. Taken together, preclinical and clinical findings provide a rationale for using FDG and FLT PET imaging for early prediction of response to therapeutics targeting the HER family.

Here, we investigated the ability of small animal FDG and FLT PET/CT imaging to predict the therapeutic response of a novel mAb mixture, Pan-HER, which comprises two EGFR-, two HER2- and two HER3-targeting mAbs. The effect of targeting all three receptors simultaneously by Pan-HER was compared with that of targeting each receptor individually.

## RESULTS

### Pan-HER inhibits tumor growth *in vivo*

**Figure 1:**
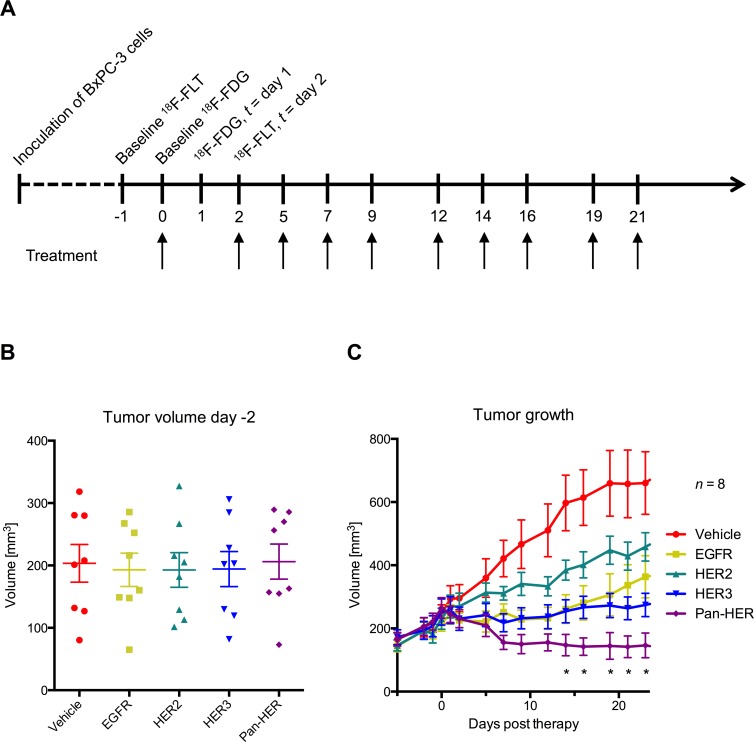
Experimental design and treatment efficacy **A.** Overview of the timing of the imaging sessions and therapy dosage. **B.** All groups had equal tumor volume at the time of the first imaging session. **C.** Pan-HER effectively inhibited tumor growth. The tumor growth inhibition in the Pan-HER group was greater compared to all of the other treatment groups (*p* ≤ 0.05).

Treatment with the different antibody mixtures was initiated when the average tumor volume reached 200 mm^3^. The mean tumor volume for all groups was equal at initiation of therapy (Figure 1B). In line with previously reported results, the Pan-HER antibody mixture effectively inhibited tumor growth in the BxPC-3 subcutaneous xenograft mouse model (Figure 1C) [1]. From day 14 and onwards, the tumor volume in the Pan-HER group was significantly smaller (*p* < 0.05) than that of the control group or any of the groups receiving antibody mixtures targeting EGFR, HER2 or HER3 individually.

### Tumor metabolism measured by FDG PET is reduced shortly after Pan-HER therapy

**Figure 2:**
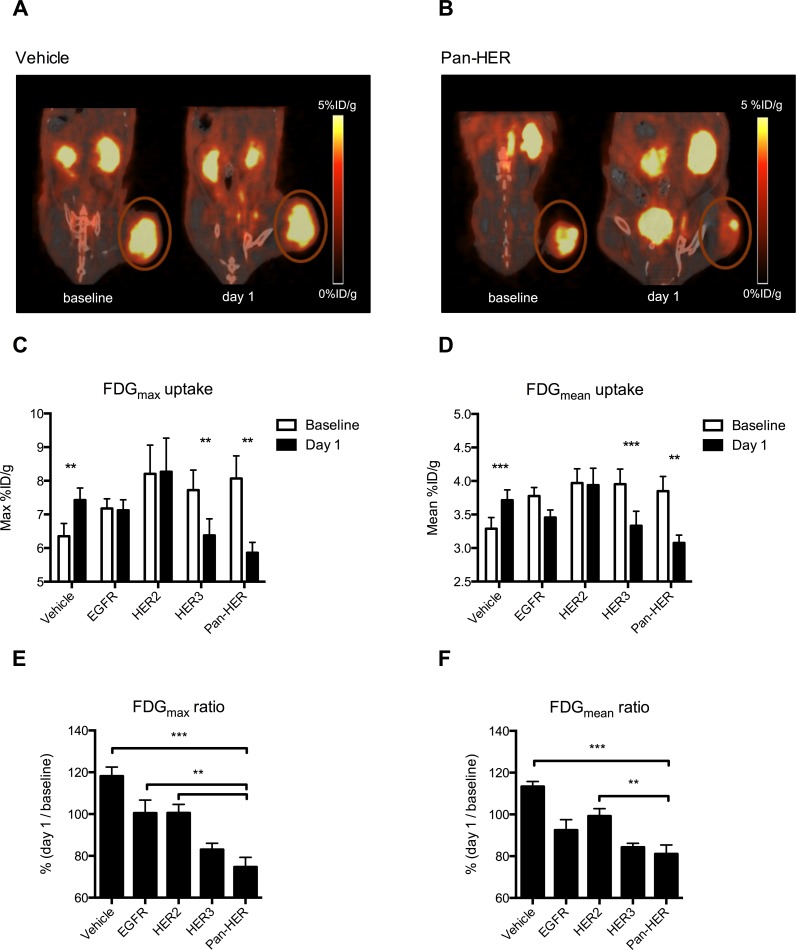
FDG uptake is reduced upon treatment with Pan-HER **A.** & **B.** Representative FDG PET/CT images of a vehicle and a Pan-HER treated mouse at baseline and one day after initiation of therapy. The tumor uptake of FDG is markedly reduced at day 1 in the Pan-HER treated mouse whereas no difference is seen for the vehicle treated mouse. **C.** & **D.** Quantitative analyses of tumor FDG uptake expressed as FDG_max_ and FDG_mean_. The FDG_max_ and FDG_mean_ uptake were significant lower at day 1 compared to baseline in the groups treated with HER3 and Pan-HER (*p* ≤ 0.01). In contrast, an increase in FDG uptake was observed in the vehicle treated group (*p* ≤ 0.01). **E.** & **F.** The FDG_max_ ratio is significantly reduced after one day of treatment in the Pan-HER group compared to the vehicle, EGFR and HER2 groups (*p* ≤ 0.01) and the FDG_mean_ ratio is significant reduced in the Pan-HER group compared to the vehicle and HER2 groups (*p* ≤ 0.01).

Small animal PET/CT imaging with FDG was performed on the day of initiation of therapy (day 0) and was repeated after one treatment dose (day 1). Tumors were FDG-positive at baseline with good tumor to normal tissue contrast. A visual reduction in FDG uptake was evident at day 1 compared with baseline for Pan-HER treated animals (Figure 2A & 2B). FDG_max_ within the tumor region of interests (ROIs) was 7.5 ± 0.3 percentage-injected dose per gram tissue (%ID/g) (range 4.6-12.6 %ID/g) and FDG_mean_ uptake within the ROIs was 3.8 ± 0.1 (range 2.85-5.2 %ID/g) at baseline.

A significant (*p* ≤ 0.001) increase in tumor metabolism, as measured by FDG PET, was observed for the mice treated with vehicle (Table 1) (Figure 2C & 2D). In contrast, a single therapeutic dose effectively reduced tumor metabolism of the mice treated with anti-HER3 (*p* ≤ 0.001) and Pan-HER (*p* ≤ 0.01) (Table 1). No difference in tumor metabolism was seen for the mice treated with EGFR and HER2 antibody mixtures (Figure 2C & 2D).

**Table 1:** FDG uptake after initiation of therapy

	FDG_max_ uptake (%ID/g)			FDG_mean_ uptake (%ID/g)		
	baseline	day 1	*p*-value	baseline	day 1	*p*-value
Pan-HER	8.1±0.7	5.9±0.3	≤0.01	3.9±0.2	3.1±0.1	≤0.01
EGFR	7.2±0.3	7.1±0.3	Ns	3.8±0.1	3.5±0.1	ns
HER2	8.2±0.9	8.3±1.0	Ns	4.0±0.2	3.9±0.3	ns
HER3	7.7±0.6	6.4±0.5	≤0.01	4.0±0.2	3.3±0.2	≤0.001
Vehicle	6.4±0.4	7.4±0.4	≤0.01	3.3±0.2	3.7±0.2	≤0.001

* non-significant (ns)

Inter-group analysis of the FDG_max_ uptake at day 1 relative to baseline showed that one dose of Pan-HER therapy caused a significantly greater reduction in tumor metabolism than treatment with the vehicle (*p* ≤ 0.001), EGFR (*p* ≤ 0.01) or HER2 (*p* ≤ 0.01) antibody mixtures alone. No statistically significant difference was seen for the mice treated with the HER3 antibody mixture *vs*. Pan-HER (Figure 2E). FDG_mean_ uptake at day 1 relative to baseline showed a significant reduction in tumor FDG_mean_ uptake in the Pan-HER treated mice compared with the mice treated with vehicle (*p* ≤ 0.001) or anti-HER2 (*p* ≤ 0.01) (Figure 2F).

### Tumor proliferation measured by FLT PET is reduced shortly after Pan-HER therapy

**Figure 3:**
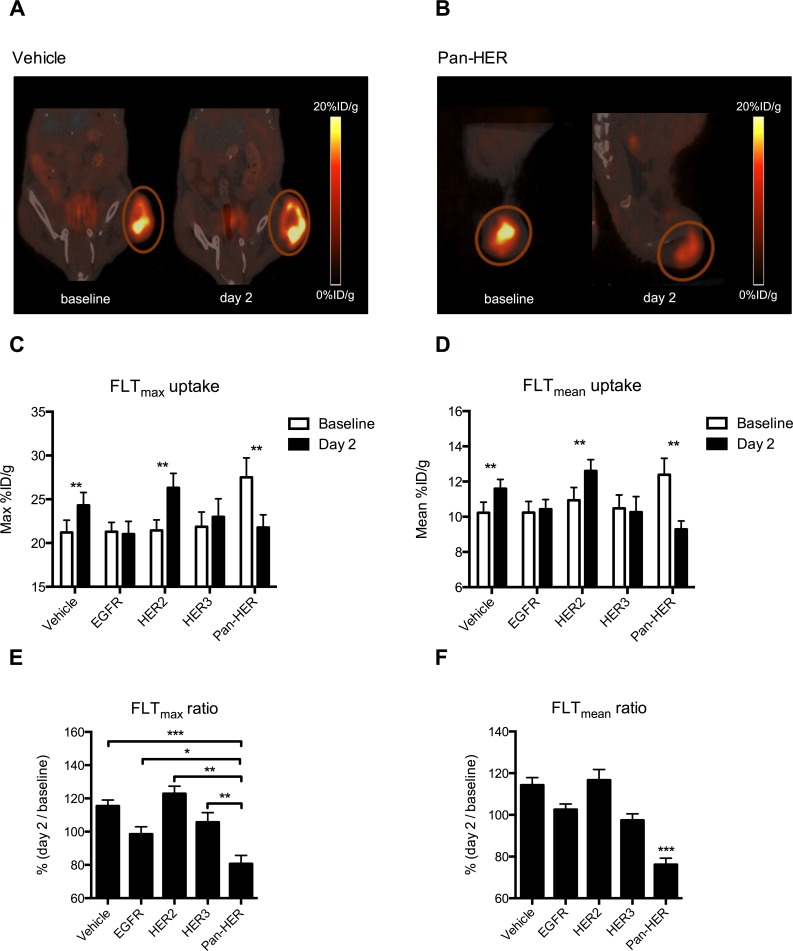
FLT uptake is reduced upon treatment with Pan-HER **A.** & **B.** Representative FLT PET/CT images of a vehicle and a Pan-HER treated mouse at baseline and two days after initiation of therapy. The tumor uptake of FLT is markedly reduced at day 2 in the Pan-HER treated mouse whereas no difference is seen for the vehicle treated mouse. **C.** & **D.** Quantitative analyses of tumor FLT uptake expressed as FLT_max_ and FLT_mean_. The FLT_max_ and FLT_mean_ uptake were significant lower at day 2 compared to baseline in the Pan-HER group (*p* ≤ 0.01). In contrast, an increase in FLT uptake was observed in the vehicle and HER2 treated groups (*p* ≤ 0.01). **E.** & **F.** The FLT_max_ and FLT_mean_ ratios are significantly reduced after two days of treatment in the Pan-HER group compared all of the other groups (*p* ≤ 0.05 and *p* ≤ 0.001).

Mice were imaged with FLT PET/CT one day before (baseline) and again two days after initiation of therapy. The baseline PET images showed high uptake of FLT at baseline with high contrast to surrounding tissues (Figure 3A & 3B). The images also showed high FLT_max_ uptake of 22.7±0.8 %ID/g (range 15.4-36.2 %ID/g) and FLT_mean_ uptake of 10.9±0.3 %ID/g (range 7.2-17.1 %ID/g) within the tumor ROIs. Visual comparison of the tumors at day 2 compared with baseline showed reduced FLT uptake in mice treated with the Pan-HER antibody mixture.

One dose of therapy was able to reduce tumor proliferation measured *in vivo* by FLT PET on day 2 in the mice treated with the Pan-HER antibody mixture compared with the baseline scan (Figure 3C & 3D). Tumor FLT uptake was significantly reduced in the Pan-HER group (*p* ≤ 0.01) while it was significantly increased in the groups treated with vehicle (*p* ≤ 0.01) and anti-HER2 (*p* ≤ 0.01) (Table 2). No differences in FLT uptake on day 2 compared with baseline were seen in the groups receiving the EGFR and HER3 antibody mixtures.

**Table 2:** FLT uptake after initiation of therapy

	FLT_max_uptake (%ID/g)			FLT_mean_ uptake (%ID/g)		
	Baseline	day 2	*p*-value	baseline	day 2	*p*-value
Pan-HER	27.5±2.2	21.8±1.4	≤0.01	12.4±0.9	9.3±0.5	≤0.01
EGFR	21.3±1.1	21.0±1.4	ns	10.2±0.6	10.4±0.5	ns
HER2	21.5±1.2	26.3±1.7	≤0.01	10.9±0.7	12.6±0.6	≤0.01
HER3	21.9±1.7	23.0±2.1	ns	10.5±0.8	10.3±0.9	ns
Vehicle	21.2±1.4	24.3±1.5	≤0.01	10.2±0.6	11.6±0.5	≤0.01

* non-significant (ns)

Non-invasive assessment of tumor proliferation by FLT PET imaging showed that one dose of Pan-HER reduced FLT_max_ uptake to 80.7% (Figure 3E & 3F) and compared with all other groups, the relative FLT_max_ and FLT_mean_ ratios were significantly reduced (*p* ≤ 0.05 and *p* ≤ 0.001, respectively).

### Change in FDG and FLT uptake shortly after therapy initiation predicts tumor growth

**Figure 4:**
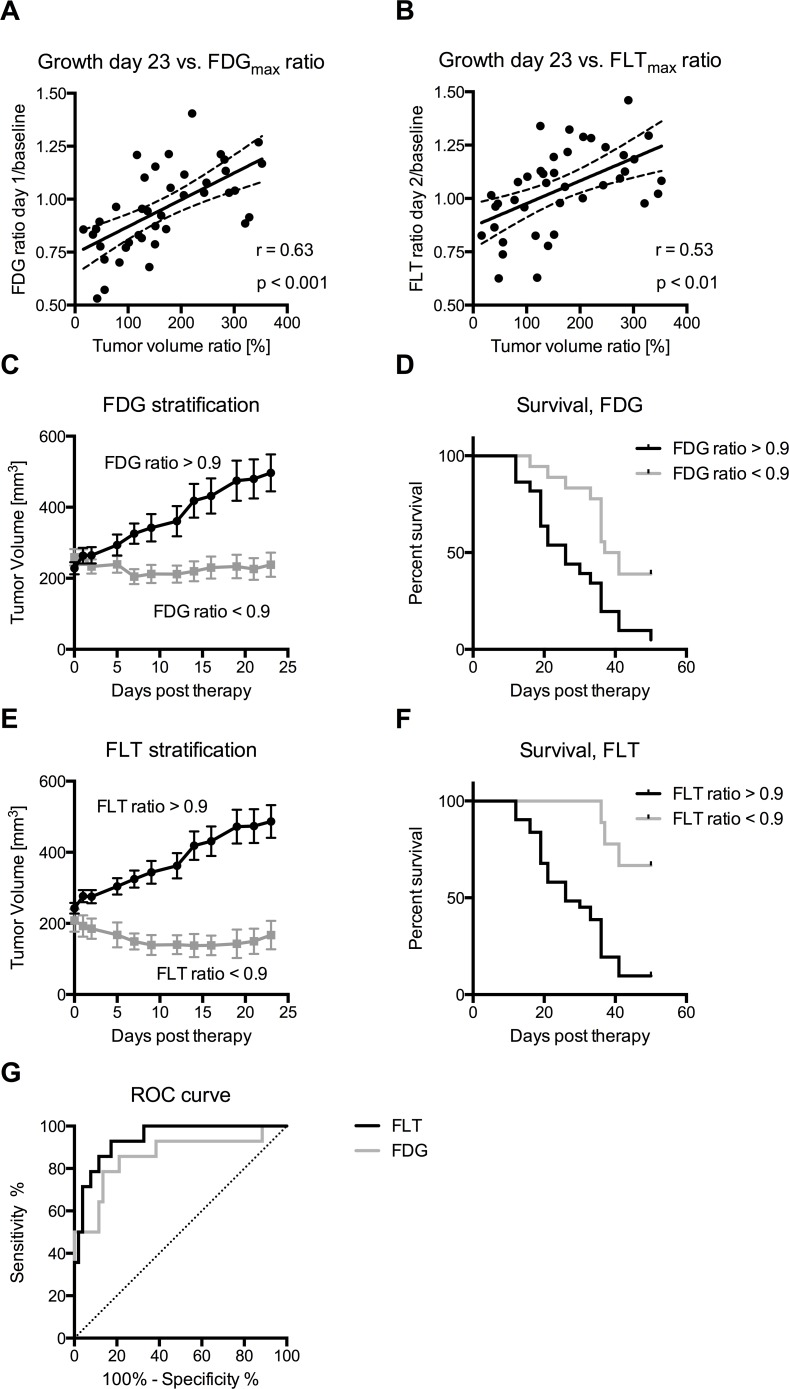
Change in FDG and FLT after therapy predicts treatment outcome **A.** & **B.** The relative tumor growth until day 23 compared to day 0, is positively correlated with the FDG_max_ and FLT_max_ ratios (*p* ≤ 0.01). **C.** & **D.** Stratification of all the mice based on their FDG_max_ ratio shows that tumor growth is inhibited in the stratification group with a FDG_max_ ratio below 0.9. Mice with a FDG_max_ ratio below 0.9 had a significant longer median overall survival (26 days versus 39 days, *p* ≤ 0.01). **E.** & **F.** Stratification of all the mice based on their FLT_max_ ratio shows that tumor growth is inhibited in the stratification group with a FLT_max_ ratio below 0.9 which also translate into improved overall survival with more than 50% of the mice being alive 50 days post therapy (*p* ≤ 0.001). **G.** ROC curves for FDG and FLT in predicting stable disease. No difference was seen in the area under the curve, which was 0.86 ± 0.07 and 0.94 ± 0.04 for FDG and FLT, respectively (*p* ≤ 0.3).

The change in tumor FLT and FDG uptake early after initiation of therapy compared with baseline carries information on future tumor growth (Figure 4). Positive correlations were seen between the change in tumor volume (day 23/day 0) and FDG_max_ (day 1/baseline), as well as FLT_max_ uptake (day 2/baseline) (Figure 4A & 4B). The correlation was slightly higher for the FDG_max_ ratio (*r* = 0.63, *p* ≤ 0.001) compared with the FLT_max_ ratio (*r* = 0.53, *p* ≤ 0.01). Correlations between tumor volume and FDG_mean_ and FLT_mean_ uptake are shown in Supplementary Figure 1.

The change in FDG_max_ uptake (day 1/baseline) can be effectively applied to stratify the tumors into subgroups that respond to the given therapy. Tumor growth in mice that had a tumor FDG_max_ ratio < 0.9 was significantly inhibited compared with that in mice with a tumor FDG_max_ ratio >0.9 (Figure 4C). Stratification on the change in FDG uptake also resulted in improved survival (*p* ≤ 0.01, Log-rank test), with a median survival of 39 days compared to 26 days after initiation of therapy for the two groups (Figure 4D).

In line with the FDG data, stratification based on the change in FLT_max_ uptake (day 2/baseline) also showed reduced tumor growth for the group of mice with FLT_max_ ratio < 0.9 compared with those with a FLT_max_ ratio >0.9 (Figure 4E). Overall survival was also improved for the mice with tumor FLT_max_ ratio < 0.9 (*p* ≤ 0.001, Log-rank test), with more than 50% of the mice with a FLT_max_ ratio < 0.9 being alive 50 days post therapy at the time of censoring (Figure 4F). Receiver operating characteristics (ROC) curves were generated to investigate the accuracy of FDG and FLT to monitor the therapeutic response (Figure 4G). The performance of FLT and FDG was comparable (*p* ≤ 0.3). For a cut-off ratio of 0.9 we obtained a sensitivity of 85.7% and 57.1% with a specificity of 76.9% and 96.2% for FDG and FLT, respectively.

### HER family receptors are down-regulated after a single dose of Pan-HER

**Figure 5:**
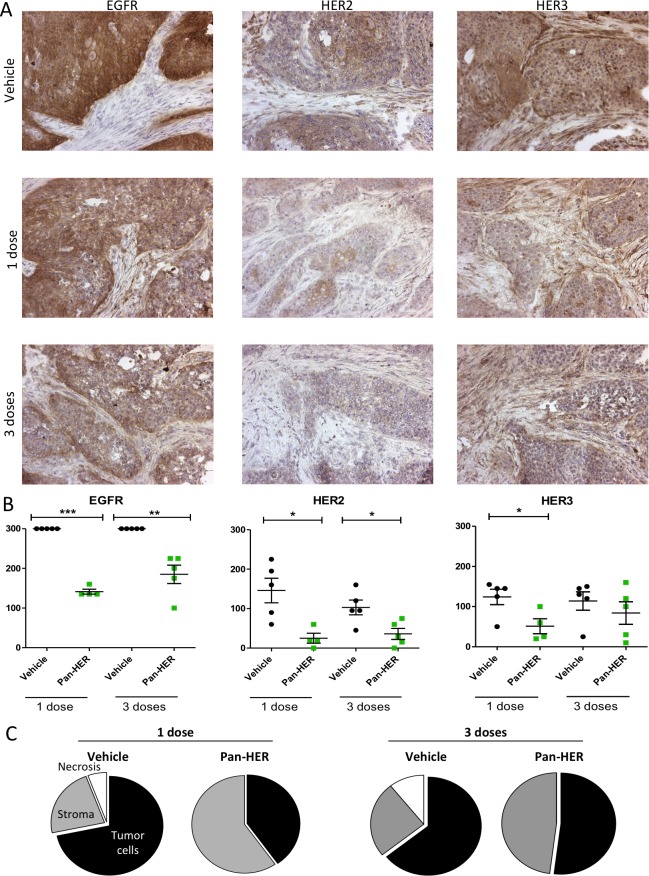
EGFR, HER2 and HER3 staining in BxPC-3 xenograft tumors after Pan-HER treatment BxPC-3 xenograft tumors were harvested after one or three doses of Pan-HER and immediately snap-frozen in liquid nitrogen. Immunohistochemistry was carried out on cryo-sections and protein expression was assessed in five tumors per treatment group. **A.** Detection of EGFR, HER2 and HER3 in BxPC-3 xenograft tumors after Pan-HER treatment. Representative images of EGFR, HER2 and HER3 staining in BxPC-3 xenograft tumors treated with a single dose of vehicle (top three images), a single dose of Pan-HER (middle three images) or three doses of Pan-HER (bottom three images). **B.** Scoring of EGFR, HER2 and HER3 staining. Protein expression was scored in five tumors per treatment group. For EGFR, HER2 and HER3 receptors, staining was scored as negative, weak staining (1+), moderate staining (2+) or strong staining (3+), multiplied by the percentage of positive cells. Each point represent the score for one tumor, bars represent means ± SEM. Statistically significant differences between Pan-HER and vehicle treated groups was assessed by Student's unpaired t-test (***:*p* < 0.0001; **:*p* < 0.01, *p < 0.05). **C.** Pan-HER treatment effect on tissue histology. Pie-charts depicting relative distribution of stromal elements, necrotic areas and adenocarcinoma (tumor cells) in the xenografts. BxPC-3 xenograft tumors were harvested after one (left panel) or three (right panel) doses of 50 mg/kg Pan-HER, and immediately snap-frozen in liquid nitrogen. Histological assessment was carried out by hematoxylin and eosin (H&E) staining of cryo-sections, and subsequently five tumors from each treatment group were assessed for stromal cell infiltration, necrotic areas and fraction of BxPC-3 adenocarcinoma cells.

**Figure 6:**
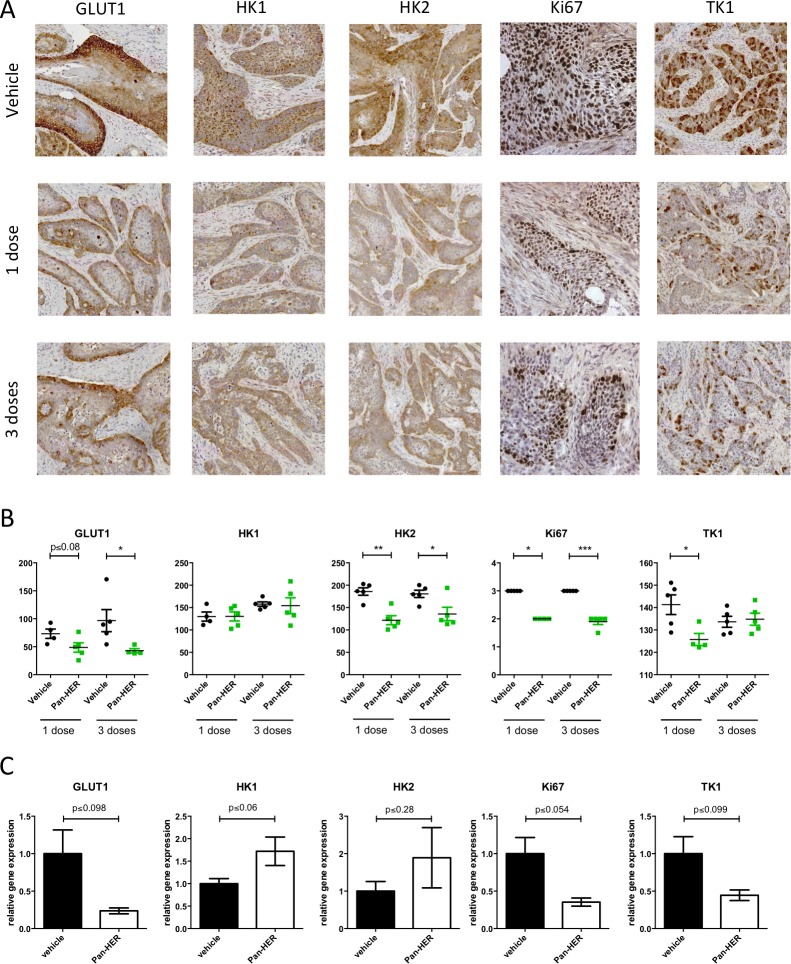
GLUT1, HK1, HK2, Ki67 and TK1 staining in BxPC-3 xenograft tumors after Pan-HER treatment BxPC-3 xenograft tumors were harvested after one or three doses of Pan-HER and protein expression was assessed in five tumors per treatment group. **A.** Detection of GLUT1, HK1, HK2, Ki67 and TK1 in BxPC-3 xenograft tumors after Pan-HER treatment. Representative images of GLUT1, HK1, HK2, Ki67 and TK1 staining in BxPC-3 xenograft tumors treated with a single dose of vehicle (top images), a single dose of Pan-HER (middle images) or three doses of Pan-HER (bottom images). **B.** Scoring of GLUT1, HK1, HK2, Ki67 and TK1 IHC staining. Protein expression was scored in five tumors per treatment group. Ki67 staining was scored as no positive cells, few, moderate number, or high number positive cells. Each point represents the score for one tumor, bars represent means ± SEM. Statistically significant differences between Pan-HER and vehicle treated groups were assessed by Student's unpaired *t*-test (***:*p* < 0.0001; **:*p* < 0.01, *p < 0.05). **C.** Gene expression of Ki67, TK1, GLUT1, HK1 and HK2. BxPC-3 xenograft tumors were harvested day 2 after one dose of Pan-HER or vehicle and immediately transferred to RNA later. Total RNA was isolated and reverse transcribed into cDNA. Relative expression of GLUT1, HK1, HK2, Ki67 and TK1 were measured with qPCR in 3-4 tumors per treatment group. The level of the gene of interest was normalized to the geometric means of two reference genes PPIA and RPLP. The data are presented as means±SEM.

In a separate experiment, vehicle and Pan-HER treated animals (*N* = 5 mice/group) were euthanized after one (day 2) and three doses (day 7) of Pan-HER therapy to investigate the early effects of therapy on target modulation (Figure 5) and biomarkers associated with proliferation and glucose metabolism (Figure 6).

Tumor growth was reduced after a single dose of Pan-HER compared to the vehicle treated group (Supplementary Figure 2). Tumor sections stained for EGFR, HER2 and HER3 showed markedly reduced receptor expression after only one dose of Pan-HER treatment compared with vehicle treated tumor sections. No further receptor down-regulation was observed in tumors obtained after three doses of Pan-HER treatment (Figure 5A & 5B). In addition, Pan-HER treatment had a profound effect on tumor histology (Figure 5C). Compared with vehicle treated tumors, one dose of Pan-HER effectively reduced the area containing tumor cells, with a resulting increase of the stromal component of the tumor sections (Figure 5C). Similar effects were observed for tumor sections obtained after three doses of Pan-HER therapy.

### Changes in FDG and FLT uptake are supported by decreased expression of underlying biomarkers after Pan-HER treatment

Protein expression of markers associated with FDG and FLT uptake are shown in Figure 6A & 6B. For the FDG markers, hexokinase 2 (HK2) was significantly reduced in the Pan-HER group after both 1 (*p* ≤ 0.01) and 3 doses (*p* ≤ 0.05) whereas glucose transporter 1 (GLUT1) was significantly reduced after 3 doses (*p* ≤ 0.05). No difference in protein expression was observed for hexokinase 1 (HK1). Significant down-regulation of the proliferation markers Ki67 and thymidine kinase 1 (TK1) was observed after 1 dosage in the Pan-HER group (*p* ≤ 0.05). Three doses of Pan-HER did not further down-regulate the proliferation markers when analyzed by IHC.

Gene expression of GLUT1, HK2, HK2, Ki67 and TK1 was measured on tumor samples on day 2 after one dose of Pan-HER and compared with gene expression in the vehicle control group. There was a clear tendency towards down-regulation of GLUT1, Ki67 and TK1 expression in the Pan-HER group as compared to the vehicle treated control group (Figure 6C & Table 3). In the case of HK1 and HK2 there was a tendency towards increased gene expression compared to the vehicle treated control group (Table 3).

**Table 3:** Gene expression of GLUT1, HK1, HK2, Ki67 and TK1

	**Pan-HER**	**Vehicle**	***p*-value**
GLUT1	0.24±0.04	1.00±0.32	0.10
HK1	1.72±0.32	1.00±0.11	0.06
HK2	1.89±0.81	1.00±0.26	0.28
Ki67	0.35±0.05	1.00±0.22	0.05
TK1	0.45±0.07	1.00±0.23	0.10

## DISCUSSION

Pan-HER, a mixture of three synergistic pairs of antibodies against EGFR, HER2 and HER3, was recently developed and demonstrated to be superior to single receptor targeting in preclinical cancer models [1]. However, along with the development of such new targeting anti-cancer therapies, it is important to develop procedures that can differentiate responders from non-responders shortly after treatment initiation. To facilitate translation into human use, such companion diagnostics should preferably be non-invasive and highly sensitive. Therefore, we have used FDG and FLT PET/CT imaging to study the early therapeutic response to both Pan-HER and antibody mixtures that target each of the receptors individually.

To investigate the use of PET for assessment of treatment effects, mice with BxPC-3 tumors were subjected to FDG and FLT PET imaging at baseline before Pan-HER treatment was initiated and again on day 1 and 2, respectively, after initiation of treatment. As shown in Figure 1C, and confirming previous findings [1], Pan-HER was more effective for treatment of BxPC-3 xenografts than targeting either of the three HER receptors individually. The superiority of simultaneous inhibition of EGFR, HER2 and HER3 compared with single targeting of these receptors was also reflected in the FDG and FLT uptake. FDG and FLT uptake were significantly decreased already at day 1 and 2, respectively, after a single dose in the Pan-HER group and changes in glucose metabolism and cell proliferation were observed before changes in tumor volume. For comparison, the tumor volume in the Pan-HER group was not significantly different from the other treatment groups until day 14.

We observed that FLT uptake was further decreased in mice receiving a single dose of Pan-HER than in mice receiving an antibody mixture targeting any of the individual receptors (Figure 3D & 3F). Similar observations were made for FDG uptake, although the differences between the Pan-HER and HER3 groups were not significant (Figure 2D & 2F). Although the differences in baseline FLT uptake between the groups were not statistically significant, the baseline FLT uptake in the Pan-HER group was higher when compared to all the other groups. The differences in baseline uptake were a result of coincidence as the mice were randomized before the PET scanning was initialized. The post-treatment (day 2) FLT uptake in the Pan-HER group was on the same level as in the EGFR and HER3 groups day 2 and, accordingly, no differences in FLT uptake between the EGFR, HER3 and Pan-HER group were observed at the post-treatment scan. This underlines the necessity for a baseline scan of each mouse as it is the intra-animal difference between pre-treatment and post-treatment FLT uptake that is predictive of further tumor growth (Figure 4).

Only minor differences in terms of level of significance were seen between measuring tracer uptake as either mean or maximum tumor uptake. This underlines the robustness of our imaging response to therapy. However, in a clinical context maximum values are preferred and has become the *de facto* standard [22]. Additionally, they generally relate more strongly to prognosis as they reflect the most aggressive phenotype of the tumor [23].

Decreased uptake of FDG and FLT has previously been reported following targeting of several members of the HER family with the small molecule inhibitors CI-1033 and PKI-166 [9-11]. Those findings are in line with the results from this study, where the Pan-HER antibody mixture targeting EGFR, HER2 and HER3 simultaneously decreased FDG and FLT uptake already at day 1 and 2 after treatment initiation. However, the changes in tracer uptake following inhibition with the two small molecule inhibitors were not observed as early as we found in the present study. FDG uptake has furthermore been used to evaluate patients with HER2 positive breast cancer, which were treated with lapatinib, a clinically approved inhibitor of EGFR as well as HER2. Pathological complete responders had a significant larger decrease in the FDG maximum standardized uptake value (SUVmax) two and six weeks post treatment initiation compared with non-responders [24].

The tumors in which FDG and FLT uptake decreased the most after the first treatment dose were the same tumors in which treatment was found to be the most effective at the end of the study. This was demonstrated by a relatively strong positive correlation between FDG and FLT uptake measured after the first therapeutic dose (day 1 and 2) and tumor volume at day 23 (Figure 4). Accordingly, FDG and FLT uptake on day 1 and 2 was able to predict future tumor growth. If this scenario translates to the clinic, it implies that patients who have the greatest decrease in FDG or FLT uptake are those in which the treatment will be most effective. We took this concept one step further and stratified all tumors on the parameter FDG or FLT ratio above or below 0.9 (Figure 4E & 4F). It was clearly demonstrated that when individual tumors were divided based on this parameter, both FDG and FLT effectively stratified the population into a group with continued tumor growth and one with tumor regression. Survival analysis showed prolonged survival of mice with FDG and FLT ratios < 0.9. This could translate into stratification of responders from non-responders in the clinical setting using a certain FDG or FLT cut-off ratio, e.g. 0.9. Especially in the case of non-responders, this would allow for a very early change from non-effective to alternative therapies. It should be noted however, that the cut-off ratio of 0.9 cannot be applied directly in a clinical setting, but has to be established clinically for the specific therapy and type of cancer.

Uptake of FDG and FLT was compared with protein and gene expression of biomarkers related to underlying mechanisms of FDG and FLT uptake (Figure 6). Expression of GLUT1 has in several studies showed to be positively correlated with FDG uptake in tumor samples and hexokinases are responsible for the phosphorylation and thereby intracellular trapping of FDG [25]. The FDG uptake in tumors was decreased after treatment with one dose of Pan-HER and we wanted to investigate if the decrease in FDG uptake was accompanied by significant decrease in GLUT1 and the hexokinases 1 and 2 measured by IHC. Two days after one single dose of Pan-HER, there was a clear tendency towards decrease in gene expression of GLUT1 in the treatment group compared to control. However, this change in expression of GLUT1 was not statistically significant. Gene expression of HK1 and HK2 did not differ significantly between the Pan-HER and control group. However, there was a trend for the gene expression to increase in the Pan-HER treatment group compared with the control. It should be kept in mind that the level of mRNA does not necessarily reflect protein expression and function [26, 27] and the differences in gene expression, protein expression, and FDG uptake could be due to post-transcriptional and translational modifications. Hence, the non-significant increase in gene expression of HK1 and HK2 could be due to such modifications or a feedback mechanism related to the Pan-HER treatment.

In order to evaluate the decrease in cell proliferation measured *in vivo* with FLT PET, expression of the proliferation marker Ki67 and TK1 was analyzed. Ki67 is a widely applied marker of cell proliferation and TK1 is the protein responsible for phosphorylation and thereby intracellular trapping of FLT [25]. Protein expression of Ki67 and TK1 were significantly decreased in the Pan-HER compared with the control group after one dose of Pan-HER. Interestingly, there was no further decrease in Ki67 and TK1 staining between tumors receiving three doses compared with tumors receiving one dose of Pan-HER. There was a clear trend for decreased gene expression of both Ki67 and TK1 in the Pan-HER compared with the control group, although this difference was not statistically significant. The non-invasive measurement of changes in cell proliferation by FLT PET was thus supported by *ex vivo* assessment of biomarkers related to cell proliferation, confirming that change in FLT uptake is a measure of change in tumor cell proliferation following Pan-HER targeting.

Expression of target receptors EGFR, HER2 and HER3 was analyzed on day 2, after one treatment dose, and on day 7, after three treatment doses of Pan-HER. The expression of all three receptors EGFR, HER2 and HER3 was significantly decreased after one dose of Pan-HER mixture. It has previously been shown that the Pan-HER mixture effectively suppresses the expression of EGFR, HER2, and HER3 after 10 doses [1]. However, it is interesting that expression of target receptors was decreased as early as two days after treatment initiation. This confirms that a therapeutic response is measurable at the molecular level after one dosage, and supports the use of PET imaging with FDG and FLT for early therapy response monitoring.

In this study of a novel Pan-HER anti-cancer therapy, we demonstrated that early imaging with both FDG and FLT PET could effectively predict long-term treatment outcome. This holds great promise for clinical translation, where FDG or FLT PET could be used as non-invasive companion diagnostics for early stratification of patients into responders or non-responders. Especially in case of non-response, early change of therapeutic strategy is possible. When performing controlled randomized clinical studies, response of FDG or FLT PET can be used as an inclusion criterion, increasing the likelihood of successful clinical outcomes.

## MATERIALS AND METHODS

### Cell culture and human pancreatic cancer xenograft model

BxPC-3 cells (ATCC CRL-1687, LGC Standards) were cultured in RPMI-1640 medium supplemented with 10% fetal bovine serum and 1% penicillin-streptomycin (Invitrogen) at 37°C and 5% CO_2_. Cells were tested negative for mycoplasma and a panel of murine pathogens. Cells in their exponential growth phase were harvested by trypsinization at 80-90% confluence and resuspended in complete medium. Tumors were generated in female NMRI nude mice (Taconic) on the flank above the hind limb by subcutaneous injection of 5 × 10^6^ cells in 100 μL. Tumor growth was monitored twice a week by caliper measurements and tumor volume was calculated using the formula: 0.52 x length x (width)^2^. All animal experiments were performed under a protocol approved by the Danish National Animal Experiments Inspectorate.

### Pan-HER and antibodies against EGFR, HER2 and HER3

Pan-HER and the antibody pairs that comprise the mixture were described previously [1]. All antibody mixtures were prepared by mixing equal amounts of the constituent antibodies.

### Pan-HER therapy and small animal PET/CT imaging

Mice were randomized into five groups (*N* = 8 mice/group) when the average tumor volume was 200 mm^3^ and therapy was initiated two days later. Antibodies were administered intraperitoneally at 50 mg/kg total antibody three times weekly for a total of 10 doses. The final endpoint was survival defined by humane endpoints: tumor mass ≥ 1g, ulceration of tumor, weight loss of more than 10% or signs of distress.

Small animal PET/CT imaging was performed with FLT and FDG at day -1/0 (baseline) and repeated on day 1 and 2 after initiation of therapy (Figure 1A). FDG and FLT were acquired from daily productions for clinical use (Rigshospitalet, Denmark).

Small animal PET imaging was performed on a microPET Focus 120 scanner. Static images were acquired for 480 s, 60 min after intravenous injection of ∼9.3 MBq (8.45-10.0 MBq) FDG or FLT. Animals were anaesthetized by sevoflurane during injections and imaging sessions. Data was acquired in list-mode and the images were reconstructed using the 3D maximum a posteriori algorithm with a voxel size of 0.3 × 0.3 × 0.8 mm and a spatial resolution at the center of the field of view of 1.2 mm. No attenuation correction was applied.

CT images were acquired on a MicroCAT II tomograph (Siemens Medical Systems) after PET imaging. Images were acquired at 360 views with 370 ms exposure at 500 μA and 70 kVp and reconstructed with an isotropic voxel size of 90 μm.

All images were analyzed offline using Inveon software (Siemens Medical Solution). PET and CT images were co-registered and ROIs drawn over the tumors to quantify the uptake of FDG or FLT expressed as percentage-injected dose per gram tissue (%ID/g). For each tumor ROI two measures were calculated: mean uptake (FDG_mean_/FLT_mean_) and maximum uptake (FDG_max_/FLT_max_) within the tumor ROIs. Survival data was stratified on the FDG_max_ ratio at day 1/baseline or the FLT_max_ ratio at day 2/baseline. A cut-off value of 0.9 was chosen as a robust measure of reduced FDG or FLT uptake at day 1 or day 2 compared to baseline. Tumor growth data was classified as stable disease or regression if the ratio of tumor volume at day 21/tumor volume at day 0 was below 1.1. ROC curves were generated for the FDG_max_ and FLT_max_ ratios.

### Immunohistochemistry

In a separate experiment, tumors were harvested after one (day 2) or three (day 7) doses of Pan-HER and immediately snap-frozen in liquid nitrogen. Protein expression was assessed in five tumors per treatment group. The immunostaining protocol for detection of EGFR, HER2, HER3 and Ki67 was previously described in detail [1]. The following primary antibodies were used: rat anti-EGFR (Abcam, clone: ICR10), rabbit anti-C-Erb-2 (DAKO), rabbit anti-Ki67 (Abcam). An in-house produced antibody (mAb5259), labeled with NHS-fluorescein (FITC) (Thermo scientific), was used for detection of HER3. The ability of the primary antibodies used for EGFR, HER2 and HER3 detection to stain tissue in the presence of excess Pan-HER was verified in pre-blocking experiments (data not shown). GLUT1, HK1, HK2 and TK1 were detected with primary antibodies rabbit anti-GLUT1 (ab15309, Abcam), rabbit anti-HK1 (HPA007043, Sigma-Aldrich), rabbit anti-HK2 (C64G5, CST) and rabbit anti-TK1 (ab57757, Abcam) in paraffin embedded sections. Briefly, paraffin embedded tumors were sectioned, deparaffinized and after heat-induced epitope retrieval all slides were blocked with Peroxidase Blocker for 10 minutes and 2% BSA for 20 minutes before incubation for 1 hour with primary antibody at room temperature. All slides were incubated in EnVision^TM^ System (K4003/K4001, DAKO) before DAB and hematoxylin stainings were performed.

Assessment of general histology (judged from hematoxylin/eosin stained slides) and IHC staining intensity was performed in a blinded fashion. IHC staining was analyzed in viable tumor area and scored as negative (0), weak (1), moderate (2), or strong (3).

### Quantitative real-time polymerase chain reaction (qPCR)

Total RNA was isolated with TRI reagent^®^ following the manufacturer's instruction (Molecular Research Center Inc.) followed by DNase treatment with TURBO DNA-*free*^TM^ Kit (Life technologies^TM^). RNA was reversed transcribed using the Affinityscript QPCR cDNA Synthesis kit (Agilent Technologies). Primers were designed in Beacon Designer (PREMIER Biosoft). Primer sequences were

GLUT1-FP: 5′-catcatcttcatcccggc-3′, GLUT1-RF: 5′-ctcctcgttgcggttgat-3′, HK1-FP: 5′-cggggaggaaagcaaaat c-3′, HK1-RP: 5′-ggcaagtgaggagggatc-3′, HK2-FP: 5′-caagcacagacattaaacct-3′, HK2-RP: 5′-gacactattctcagcacaag-3′, Ki67-FP: 5′-tcccgcctgttttctttctgac-3′, Ki67-RP: 5′-ctctccaaggatgatgatgctttac-3′, TK1-FP: 5′-gccgatgttctcaggaaaaagc-3′, TK1-RP: 5′-gcgagtgtctttggcatacttg-3′, PPIA-FP: 5′-cggatttgatcatttggtg-3′, PPIA-RP: 5′-cagggaatacgtaaccag-3′, RPLP-FP: 5′-ccaggctttaggtatcac-3′ and RPLP-RP: 5′ggttgtagatgctgcc-3′.

Gene expression was quantified on an Mx3005P real-time PCR system (Stratagene) using Brilliant III Ultra-Fast SYBR^®^ Green QPCR Master Mix (Agilent Technologies). The thermal profile was 3 minutes of denaturation at 95°C followed by 40 cycles of 20 seconds denaturation at 95°C and 20 seconds of annealing/extension at 60°C. A dissociation curve was thereafter obtained by denaturation of the products for 1 minute at 95°C followed by data collection while temperature increases from 55°C to 95°C. QPCR data were analyzed in qbase+ (Biogazelle). The relative quantification of the gene of interests was presented as fold changes in the treatment group compared to the control group on day 2 normalized to the geometric mean of two reference genes. The two most stable reference genes were found from a panel of 12 candidate genes in the human reference gene panel (TATAA Biocenter AB) using the geNorm algorithm.

### Statistical analysis

Unless stated otherwise, data are expressed as means ± SEM. Student's *t*-test was applied to compare uptake values and tumor volumes at each time-point between Pan-HER and the other treatment groups without adjustment for multiple comparisons. One-way ANOVA with Bonferroni's multiple comparisons test was used to test, if the uptake ratios of FDG and FLT were different in the Pan-HER group compared to the other groups. Linear regression analyses were performed to investigate correlations. The Log-rank test was applied to analyze survival data. Statistical analyses were performed using GraphPad Prism 6.0d (GraphPad Software, Inc.).

## SUPPLEMENTARY MATERIAL FIGURES


