# Supplementary material for: In vivo imaging of therapy response to a novel Pan-HER antibody mixture using FDG and FLT positron emission tomography
Source: Oncotarget. 2015 Oct 9;6(35):37486–99. doi: 10.18632/oncotarget.6060 (PMC4741943; doi:10.18632/oncotarget.6060)
Supplement: Supplementary file 1 [file oncotarget-06-37486-s001.pdf]

In vivo imaging of therapy response to a novel Pan-HER antibody mixture using FDG and FLT positron emission tomography

Supplementary Material

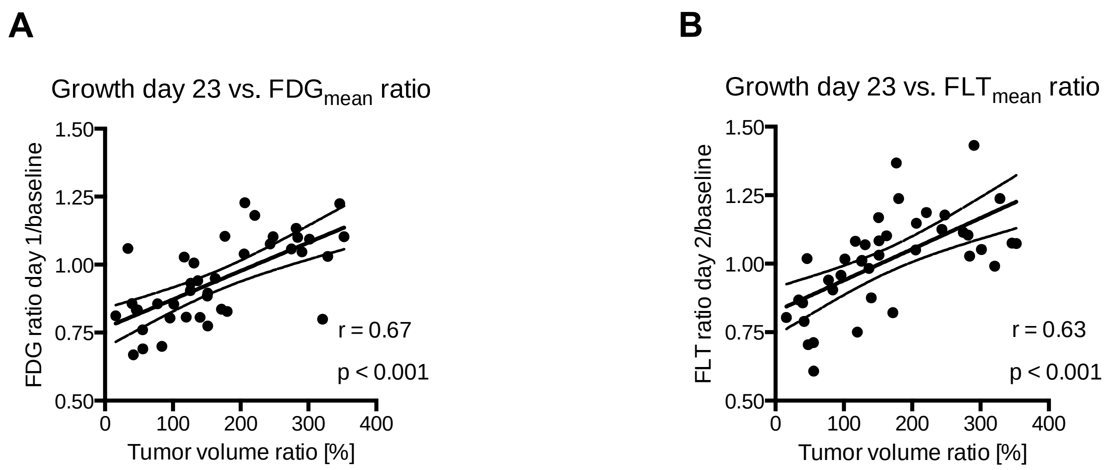

Supplementary Figure 1: Change in FDG and FLT after therapy predicts treatment outcome. The relative tumor growth until day 23 compared to day 0, is positively correlated with the FDG_{mean} ratio (**A**) and the FLT_{mean} ratio (**B**), $p \leq 0.001$.

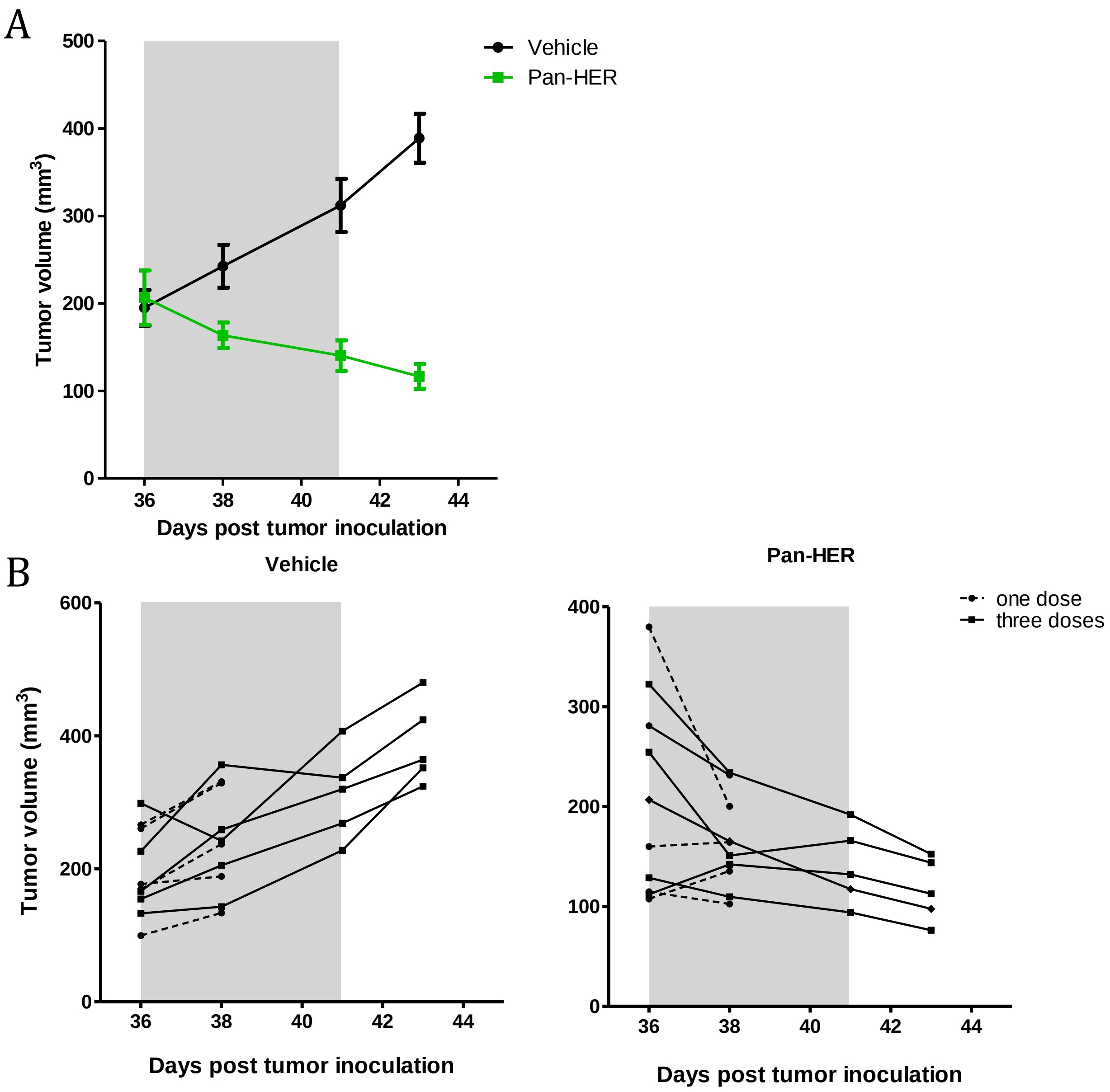

Supplementary Figure 2: Tumor volumes from mice used for *ex vivo* biomarker evaluation. A) Tumor growth curves for the Pan-HER and vehicle groups. **B)** Individual tumor volumes for mice in the vehicle group (Left) and Pan-HER group (Right).
